# Evolutionarily Conserved Long Non-coding RNA Regulates Gene Expression in Cytokine Storm During COVID-19

**DOI:** 10.3389/fbioe.2020.582953

**Published:** 2021-01-15

**Authors:** Olanrewaju B. Morenikeji, Kahleel Bernard, Ellis Strutton, Madeleine Wallace, Bolaji N. Thomas

**Affiliations:** ^1^Department of Biology, Hamilton College, Clinton, NY, United States; ^2^Department of Biomedical Sciences, Rochester Institute of Technology, Rochester, NY, United States

**Keywords:** lncRNA, cytokine storm, genes, markers, regulation, COVID-19

## Abstract

Coronavirus is a family of viruses including alpha-, beta-, gamma-, delta-coronaviruses. Only alpha- and betacoronaviruses have been observed to infect humans. Past outbreaks of SARS-CoV and MERS-CoV, both betacoronavirus, are the result of a spillover from animals. Recently, a new strain termed SARS-CoV-2 emerged in December 2019 in Wuhan, China. Severe cases of COVID-19, the disease caused by SARS-CoV-2, lead to acute respiratory distress syndrome (ARDS). One contributor to the development of ARDS is cytokine storm, an overwhelming inflammatory immune response. Long non-coding RNAs (lncRNAs) are genetic regulatory elements that, among many functions, alter gene expression and cellular processes. lncRNAs identified to be pertinent in COVID-19 cytokine storm have the potential to serve as disease markers or drug targets. This project aims to computationally identify conserved lncRNAs potentially regulating gene expression in cytokine storm during COVID-19. We found 22 lncRNAs that can target 10 cytokines overexpressed in COVID-19 cytokine storm, 8 of which targeted two or more cytokine storm cytokines. In particular, the lncRNA non-coding RNA activated by DNA damage (NORAD), targeted five out of the ten identified cytokine storm cytokines, and is evolutionarily conserved across multiple species. These lncRNAs are ideal candidates for further *in vitro* and *in vivo* analysis.

## Introduction

Coronavirus is a family of respiratory viruses including alpha-, beta-, gamma-, and delta-coronaviruses, of which only alpha- and beta-coronaviruses have been observed to infect humans (Cui et al., 2019). Past outbreaks of SARS-CoV and MERS-CoV, both betacoronaviruses, are the result of zoonotic spillover (de Wit et al., 2016). In December 2019, a new strain of coronavirus termed SARS-CoV-2 emerged in a seafood market in Wuhan, China, thought to be a zoonotic spillover from pangolins (Sun et al., 2020; Zhang et al., 2020). While SARS-CoV-2 shares a 96% sequence similarity to a horseshoe bat coronavirus, there is ongoing research to identify a more recent intermediate host species (Mallapaty, 2020; Zhou P. et al., 2020). As of October 14, 2020, over 38.4 million cases of COVID-19, the disease caused by SARS-CoV-2, have been confirmed worldwide (Dong et al., 2020). Common symptoms of COVID-19 include fever, cough, pounding headaches, anosmia and ageusia, yet one unique attribute is the role of asymptomatic individuals in the spread of disease (Guan et al., 2020; Vaira et al., 2020). Asymptomatic spread is estimated to be responsible for 40–45% SARS-CoV-2 transmission, as compared to 9.8% in MERS-CoV (Al-Tawfiq, 2020; Oran and Topol, 2020). Severe cases of COVID-19 has been shown to lead to acute respiratory distress syndrome (ARDS), the leading cause of death from COVID-19 (Ruan et al., 2020). ARDS has a mortality rate of around 75%; thus it is critical to develop a thorough understanding of this pathology, examine contributory factors, dissect possible role for host genetics in variable disease outcome and search for potential treatments (Yang et al., 2020).

One contributor to the development of ARDS is cytokine storm, a dysregulated, overwhelming pro-inflammatory immune response (Ye et al., 2020). Cytokines are the main mode of communication between innate immune cells, and are a part of a normal innate immune response that serves as the first line of defense against pathogens (Altan-Bonnet and Ratnadeep, 2019; Ye et al., 2020). However, an aggressive pro-inflammatory response can also circulate throughout the body and cause damage to tissue resulting in septic shock and multi-organ failure (Diao et al., 2020; Tay et al., 2020; Xiong et al., 2020). Multiple studies have identified cytokines upregulated in cytokine storm, including IL-2R, IL-6, IL-8, IL-10, and TNFα (Chu et al., 2020; Liu et al., 2020; Mehta et al., 2020; Qin et al., 2020). Thus, a potential treatment modality is the regulation of pro-inflammatory host immune response to COVID-19.

Currently, there are no approved antiviral treatments or vaccines specific for COVID-19 (Wu C. et al., 2020; Wu Y. et al., 2020). Current treatment consists of symptomatic and supportive care, with treatment of secondary conditions. Treatments currently in use include antivirals such as remdesivir and convalescent plasma (Chen G. et al., 2020; Chen L. et al., 2020; Chen N. et al., 2020; Chen X. et al., 2020). The current protocol for diagnosing COVID-19 is a viral RNA RT-PCR test with a nasopharyngeal or oropharyngeal sample (Beeching et al., 2020). Antibody tests using blood samples have also been developed to detect IgG and IgM antibodies indicative of a prior exposure. These tests are performed using ELISA and lateral flow immunoassays, and have the caveat of detecting cases after the first week of infection and lower sensitivity for asymptomatic individuals (Li et al., 2020; Okba et al., 2020). A diversity of vaccine candidates are currently being tested, with 115 vaccine candidates and five vaccines in phase 1 clinical trials (Thanh Le et al., 2020). Concomitant with the need for treatments tailored to COVID-19 is the need for a diverse set of diagnostic and prognostic markers related to disease severity. Markers forecasting increased disease severity can serve as indicators for specific treatments before a severe disease phenotype is observed, leading to earlier interventions and better patient outcomes. So far, raised procalcitonin (PCT) levels and low platelet counts have been found to be associated with an increased risk of severe cases of COVID-19 infection and death (Li et al., 2020; Lippi and Plebani, 2020; Lippi et al., 2020). One avenue that is yet to be explored is COVID-19 diagnostic markers designed on the basis of gene regulation. While viral RNA load has been examined as a proxy for virus titer and a predictor of disease severity, no study to date has looked at endogenous genetic regulatory elements as markers of COVID-19 disease severity. Thus, this area warrants further inspection and characterization to inform treatment and diagnostic development.

Long non-coding RNAs, or lncRNAs, are non-coding RNA strands over 200 nucleotides in length that have structural, catalytic, or regulatory roles (Yang et al., 2014). So far, numerous lncRNAs including MALAT1, SNHG14, and XIST have been identified to play roles during inflammatory immune response (Chen C. C. et al., 2018; Chen H. et al., 2018; Zhong et al., 2018; Ma et al., 2019). LncRNAs NORAD, PAAN and NRON have been demonstrated to be important in modulating viral pathogenesis in hepatitis C, influenza A and HIV-1, respectively (Imam et al., 2015; Sur et al., 2018; Wang et al., 2018). Additionally, a database of differential lncRNA expressed in mice during SARS-CoV pathogenesis through RNA-seq has been created (Josset et al., 2014), including a recent characterization of transcriptional lncRNA in normal bronchial epithelial cells, underscoring their importance in immune response regulation (Vishnubalaji et al., 2020). There has been significant progress made in the creation of tools to computationally identify and functionally annotate lncRNAs. Human ncRNA expression profiles have been created for normal tissues, cancer cell lines, and subcellular components (Djebali et al., 2012; Klijn et al., 2015; Uhlén et al., 2015). Functionally, softwares are available to predict lncRNA-RNA and lncRNA-protein interactions (Kato et al., 2010; Bellucci et al., 2011; Kiryu et al., 2011; Lu et al., 2013). Tools are also available to predict lncRNA secondary structures, consensus secondary structures, tertiary structures and joint secondary structures, and a database has also been created to compile lncRNA-target relationships from literature (Iwakiri et al., 2016; Cheng et al., 2019). However, the lncRNA transcriptome has yet to be comprehensively annotated; challenges in the area of lncRNA research include the relative low expression levels of lncRNAs, lack of understanding of the lncRNA sequence–function relationship, and weak conservation during evolution (Uszczynska-Ratajczak et al., 2018). Using publicly available lncRNA databases and computational tools, we sought to identify lncRNAs involved in COVID-19 cytokine storm and understand their role in disease pathology.

## Materials and Methods

### Identification of Significant Cytokines in COVID-19 Cytokine Storm

Cytokines associated with increased COVID-19 cytokine storm were identified using the search engines Google Scholar, PubMed, and Web of Science, and a literature review by Costela-Ruiz et al. (2020) amongst others. The literatures were retrieved and manually curated for cytokine reports in several COVID-19 patient cases. Our literature search included only publications between 2019 and 2020 capturing the period of COVID-19 outbreak. Based on the reports from published literatures, cytokines selected for further analysis were corroborated by at least 3 sources to be associated with increased COVID-19 severity and subsequent cytokine storm.

### Prediction of Cytokine-lncRNA Relationships

Cytokines identified in the previous step were queried in the LncRNA2Target, a database compiling lncRNA-target relationship, as described by Cheng et al. (2019). Briefly, cytokine official gene names were used to query against human lncRNAs database, using default settings. Sequences for identified lncRNAs were retrieved from NONCODE (noncode.org) (Zhao et al., 2016), NCBI Gene (ncbi.nlm.nih.gov/gene), and NCBI Gene Expression Omnibus (GEO) (ncbi.nlm.nih.gov/geo). In order to ascertain the binding ability of lncRNA-target interactions, we assessed a minimum free energy for each cytokine-lncRNA gene pair previously identified by LncRNA2Target. LncTar was used to calculate the normalized binding free energy (ndG) for lncRNAs and their associated cytokine genes as described (Li et al., 2015). LncRNA sequences are usually long, therefore, where too long sequences were found, to be accepted by LncTar were divided into sections around 17,500 nt in length to be run individually.

### Protein-Protein Network Analysis of Significant Cytokines in COVID-19 Cytokine Storm

To gain insight into molecular interactions between cytokines and pathogenic mechanisms, we performed a protein-protein interaction network analysis of the 10 most significant cytokines using Search Tool for the Retrieval of Interacting Genes database (STRING-DB;string-db.org, Szklarczyk et al., 2017). The gene sequences associated with the official gene names were retrieved from ensembl database (ensembl.org) and used in performing the network analysis as described (Jiang et al., 2019; Morenikeji and Thomas, 2019). In order to visualize the functional relationships between lncRNAs, cytokines targets, and metabolic pathways, a network analysis was created using Cytoscape (v3.7.2) (cytoscape.org). To identify significant nodes in the network, Molecular Complex Detection (MCODE); a Cytoscape plug-in was used to generate network clustering based on regions with dense connections (Jiang et al., 2019). We hypothesize that lncRNA that target multiple genes with the same molecular function will perturb or regulate the same pathways during the disease. The lncRNA-target-pathways network was constructed with Cytoscape (v3.7.2) program (Smoot et al., 2011).

### Pathway Enrichment, Functional Annotation, and Gene Ontology Analysis of Significant Cytokines and Identified lncRNAs Associated With COVID-19 Cytokine Storm

In order to elucidate pathways that are significantly perturbed in COVID-19 cytokine storm, GeneAnalytics (geneanalytics.genecards.org) was used to identify metabolic pathways and gene ontology terms associated with such cytokines. Likewise, to understand the role that the identified lncRNAs play in disease pathology, we used GeneAnalytics to identify associated diseases, as described (Fuchs et al., 2016). Metabolic pathways, gene ontology terms, and diseases selected for further analysis had a corrected *p*-value equal to or below 0.0001 and were associated with 4 or more cytokines. GeneAnalytics was used to identify diseases associated with the lncRNAs identified previously. Diseases selected for further analysis had a corrected *p*-value equal to or below 0.0001 and were associated with 4 or more lncRNAs.

### Evolutionary Trace of Non-coding RNA Activated by DNA Damage (NORAD), Structural Prediction and Characterization

The lncRNA NORAD (Non-Coding RNA Activated by DNA Damage) found from the previous steps was significantly predicted/reported to target more cytokine genes than any other lncRNA, so it was selected for further evolutionary trace analysis into to depict its evolutionary conservation among other species. Although, lncRNAs are known to be poorly conserved, therefore we proposed that a lncRNA targeting multiple genes with high evolutionary conservation would be a significant maker during COVID-19 cytokine storm. NCBI BLASTn was used to search for related lncRNAs in other species against the NORAD nucleotide sequence. Out of the top 100 results returned, one sequence was selected for each genus returned. A multiple sequence alignment and phylogenetic tree was created using MEGA (v7), with the neighbor-joining clustering method and bootstrapping with 500 iterations (Kumar et al., 2016). The phylogenetic tree was imported to ITOL (itol.embl.de) for proper visualization (Letunic and Bork, 2019). In addition, based on the annotated human Reference Sequence (RefSeq) of NORAD (LINC00657), we obtained its mammalian conservation across hundreds of genomic sequences and expressed sequence tags (ESTs) using UCSC Genome Browser (https://genome.ucsc.edu/cgi-bin/hgGateway). In order to gain insight into the thermodynamic stability and evolutionary conservation of lncRNA NORAD secondary structure, we carried out a multiple sequence alignment of six mammals (human, chimpanzee, monkey, rat, mouse and dog) and transferred to RNAzWebServer (http://rna.tbi.univie.ac.at/cgi-bin/RNAz/RNAz.cgi), using default parameter as described (Mathews et al., 2004; Lorenz et al., 2011). This server uses dynamic programming algorithm to determine encoding base-pair probabilities and RNA folding. A predicted RNA structure from the MSA with probability higher than 0.5 (*p* > 0.5) is considered a strong evidence for structural RNA and evolutionary conservation.

## Results

### Identification of Significant Cytokines in COVID-19 Cytokine Storm

**Figure 1:**
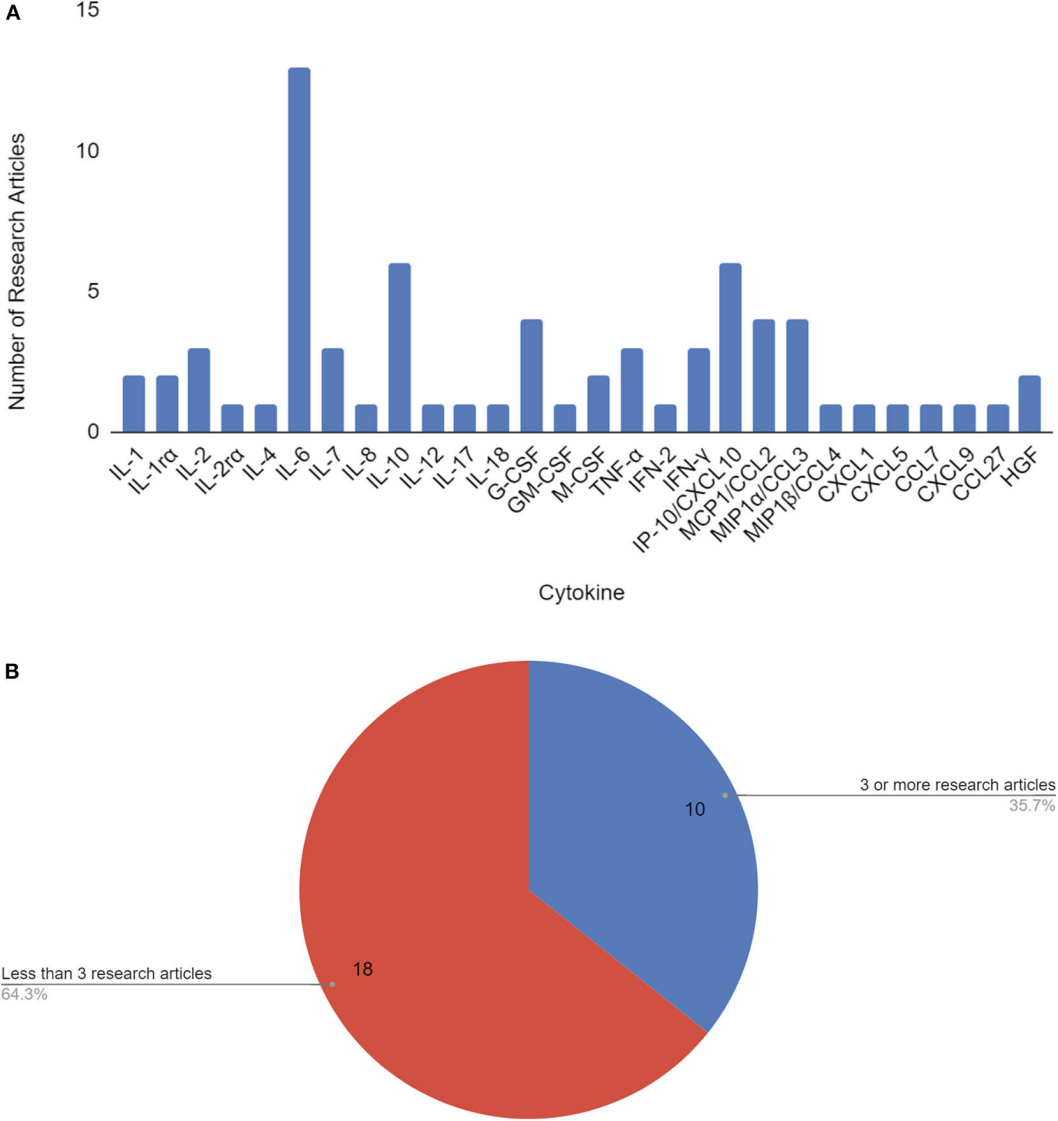
Cytokines by number of research articles identifying the cytokine to be associated with COVID-19 disease severity and/or cytokine storm **(A)**. Cytokines used in further analysis (10) were associated with 3 or more papers that cited them as correlated with COVID-19 disease severity and/or cytokine storm **(B)**.

As of June 3, 2020, 210 papers appear in Web of Science, 518 papers in PubMed, and 6,420 results in Google Scholar for COVID-19 cytokine storm. Our literature search revealed a total of 17 papers that identified 28 cytokines to be significantly involved or associated with COVID-19 cytokine storm (Figure 1A, Supplementary Table 1). To pin-point significantly reported cytokines, ten cytokines met the threshold for consideration of having 3 or more research articles citing it as being associated with COVID-19 disease severity and/or cytokine storm, and were selected for further analysis (Table 1, Figure 1B). IL-6, IL-10, and IP-10 were mentioned most often in literature as being significant, being mentioned in 13, 6, and 6 papers, respectively.

**Table 1:** COVID-19 cytokine storm significant cytokines used for further lncRNA analysis.

**Cytokine target**	**Official gene name**	**mRNA sequence accession number**
IL-2	IL-2	BC070338.1
IL-6	IL-6	BC015511.1
IL-7	IL-7	BC047698.1
IL-10	IL-10	BC104253.1
G-CSF	CSF3	BC033245.1
TNFα	TNFα	BC028148.1
IFNγ	IFNγ	BC070256.1
IP-10/CXCL10	CXCL10	BC010954.1
MCP1/CCL2	CCL2	BC009716.1
MIP1a/CCL3	CCL3	BC071834.1

*Official gene names and mRNA sequence accession number are shown*.

### Prediction of Cytokine-lncRNA Relationships

We identified a total of 24 cytokine-lncRNA relationships using LncRNA2Target (Table 2). Two lncRNAs were removed from further analysis due to identical primer sequences. Thus, 22 lncRNAs were selected for further analysis. All lncRNA-target pairs identified by LncRNA2Target had a normalized binding free energy (ndG) above −0.02 (Supplementary Table 2). The lncRNAs LNC-LBCS, STXBP5-AS1, and CDK6-AS1 were too long to run in LncTar and had to be divided into multiple sections for analysis. The lncRNA NORAD was found to pair with five out of ten of the cytokines in our study including IL-6, IL-10, CSF3, TNFα and CXCL10, more than any other lncRNAs (Table 3). Therefore, this lncRNA was selected for further evolutionary trace analysis to identify its conservation across other species.

**Table 2:** The most significant lncRNAs associated with COVID-19 cytokine storm cytokines, accompanied by lncRNA target prediction analysis using average ndG values.

**Cytokine target**	**Number of lncRNAs**	**lncRNA gene names**	**Average ndG**
IL-2	3	BANCR, lnrCXCR4, DRAIC	−0.0392
IL-6	7	lnc-IL7R*, LNCSRLR, SBF2-AS1, RAD51-AS1, LNC-LBCS, NORAD, SENCR	−0.038409091
IL-7	2	TUG1, SBF2-AS1	−0.06735
IL-10	4	GAS5, lnrCXCR4, NORAD, SNHG1	−0.0669
CSF3	2	STXBP5-AS1, NORAD	−0.045418182
TNFα	6	THRIL, RAD51-AS1, CASC15, NORAD, GAS5, NRCP*	−0.044842857
IFNγ	2	TMEVPG1, PRC1-AS1	−0.0257
CXCL10	1	NORAD	−0.0319
CCL2	5	MALAT1, TUG1, RAD51-AS1, SNHG1, NRAV	−0.04588
CCL3	3	lnrCXCR4, NRAV, CDK6-AS1	−0.067471429

*lncRNAs with an asterisk are not official gene names, and are not annotated in NCBI gene*.

*ndG; a cutoff to determine whether an RNA molecule interacts with target gene*.

**Table 3:** The most significant lncRNAs associated with COVID-19 cytokine storm cytokines, along with the lncRNA accession number.

**lncRNA gene name**	**Number of cytokine targets**	**Accession number**
NORAD	5	NONHSAT079548.2
RAD51-AS1	3	NONHSAT041865.2
lnrCXCR4	3	GSE104018
SBF2-AS1	2	NONHSAT017939.2
TUG1	2	NONHSAT084833.2
GAS5	2	NC_000001.11:c173868882-173863899
SNHG1	2	NONHSAT021826.2
NRAV	2	NONHSAT031176.2
BANCR	1	NONHSAT131775.2
DRAIC	1	NC_000015.10:69561720-69571440
lnc-IL7R*	1	AL713738.1
LNCSRLR	1	NC_000003.12:c146069185-146066344
LNC-LBCS	1	NC_000006.12:c19804759-19729421
SENCR	1	NONHSAT025072.2
STXBP5-AS1	1	NC_000006.12:c147204614-146841388
THRIL	1	NONHSAT164169.1
NRCP*	1	NR_046371.2
TMEVPG1	1	NONHSAT029277.2
PRC1-AS1	1	NC_000015.10:90966369-90988624
MALAT1	1	NC_000011.10:65497738-65506516
CDK6-AS1	1	NC_000007.14:92836483-92917187
CASC15	1	NONHSAT108049.2

*lncRNAs with an asterisk are not official gene names; not annotated in NCBI database*.

### Protein-Protein Network Analysis of Significant Cytokines in COVID-19 Cytokine Storm

**Figure 2:**
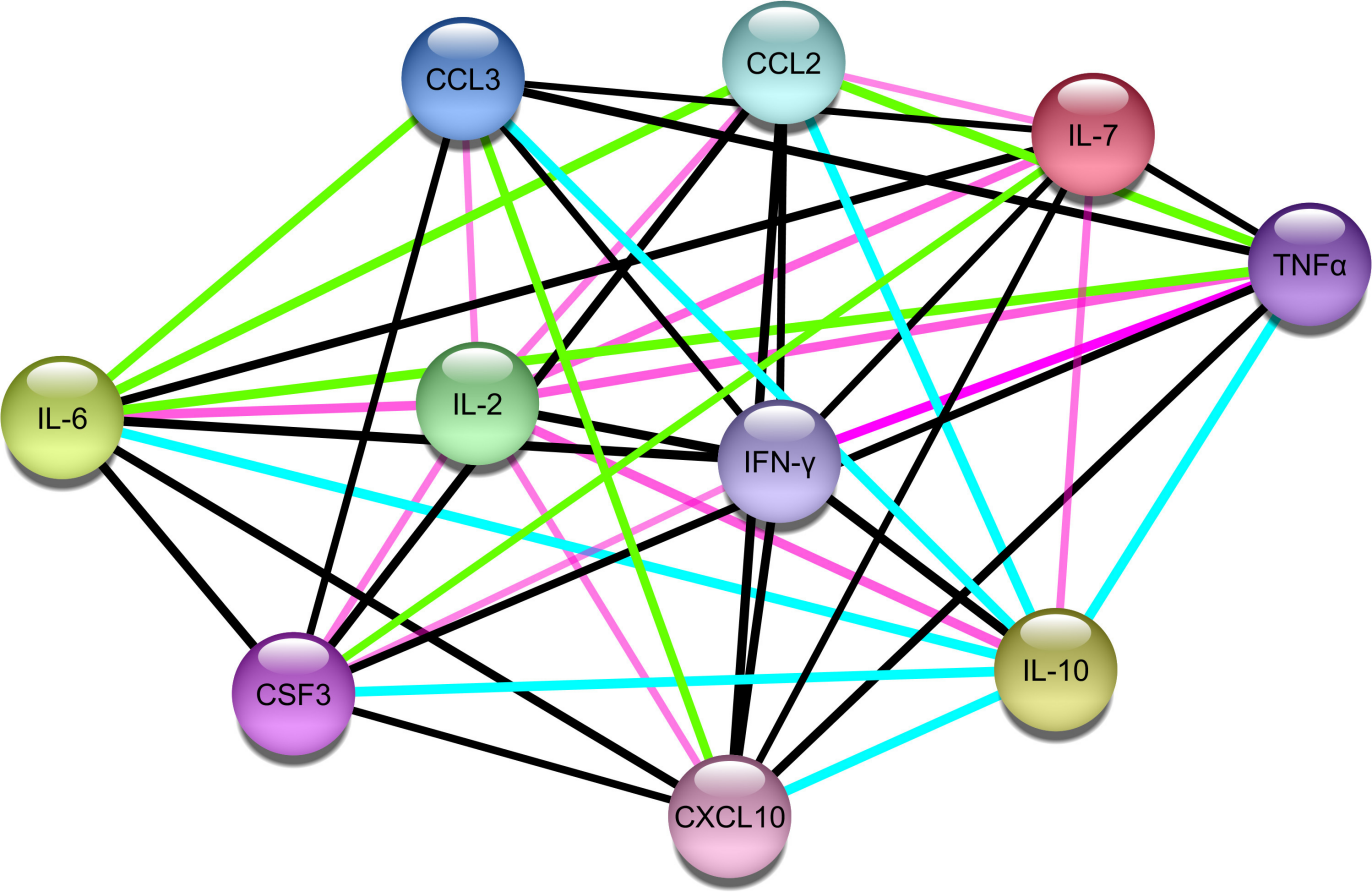
Protein-protein interaction network of the 10 cytokines associated with COVID-19 cytokine storm (Edge color legend; blue: from curated database; pink: experimentally determined; green: text mining; black: co-expression).

**Figure 3:**
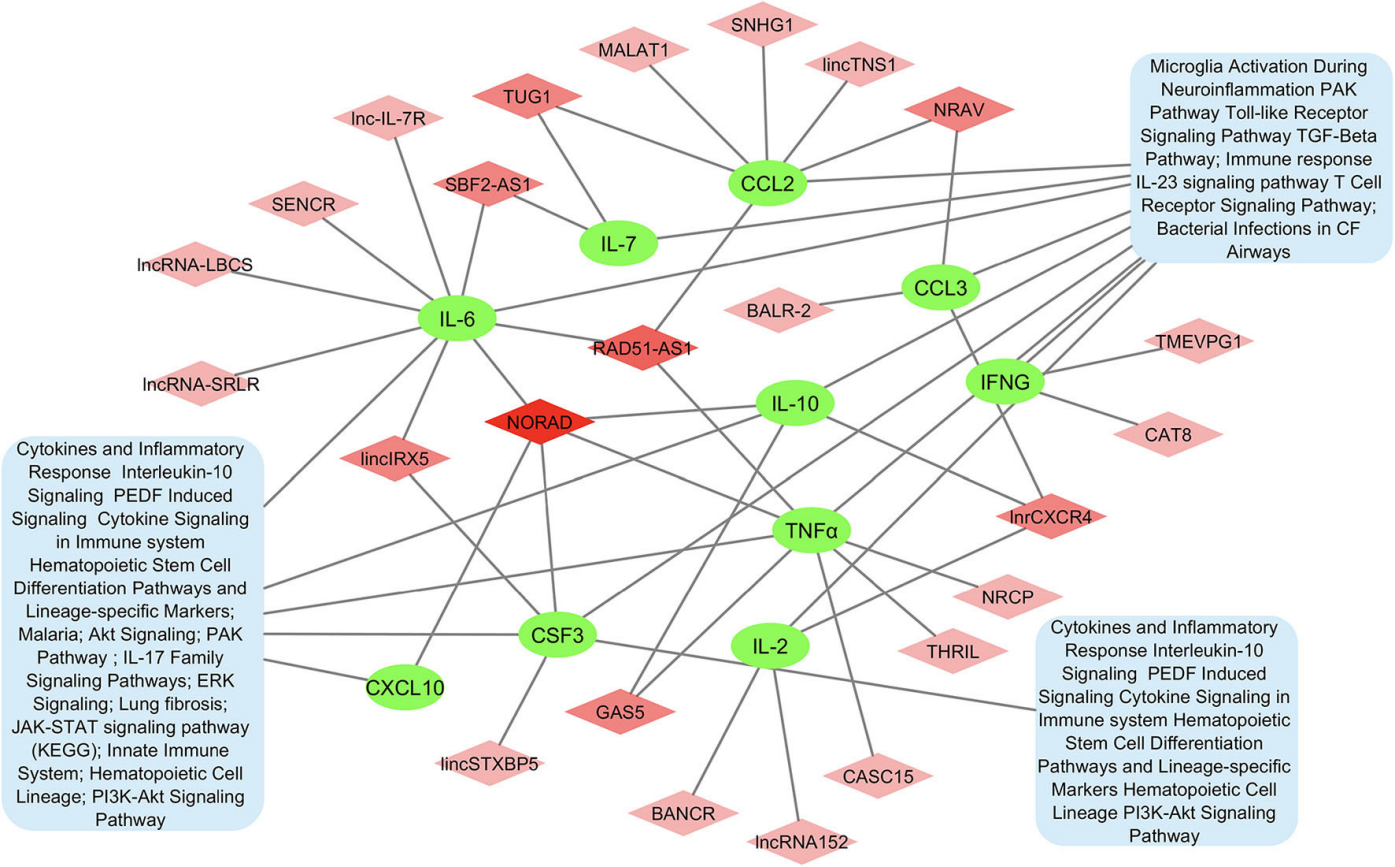
Network of lncRNA and cytokine pathway associations for most significant cytokines in COVID-19 cytokine storm. Cytokines are shown in green, lncRNAs associated with multiple cytokines are shown in darker shades of pink, and pathways are shown in blue.

A protein-protein network of the 10 most significant cytokines in COVID-19 cytokine storm was created to elucidate molecular interaction and possible mechanism for co-expression using STRING-DB (Figure 2). We found IL-2 connected to the other cytokines only through text mining, while all other cytokines are interconnected with each other through both text mining and co-expression. In addition, IL-6, TNFα, CCL3, CXCL10, and IFNγ are all connected to IL-10 through interactions identified from curated databases. TNF is experimentally determined to be connected to IFNγ. A network analysis of lncRNA and cytokine pathway associations was created using Cytoscape (Figure 3). The lncRNAs lnrCRCX4, NORAD, and RAD51-AS1 significantly target or regulate 3 or more cytokines, while GAS5, NRAV, TUG1, SBF2-AS1, and lincIRX5 lncRNAs regulate two cytokines. Three major groups of pathways were cataloged based on the number of cytokines present in the pathway; in particular, it was found that eight pathways were associated with the cytokine IL-6.

### Pathway Enrichment, Functional Annotation, and Gene Ontology Analysis of Significant Cytokines and Identified lncRNAs Associated With COVID-19 Cytokine Storm

**Figure 4:**
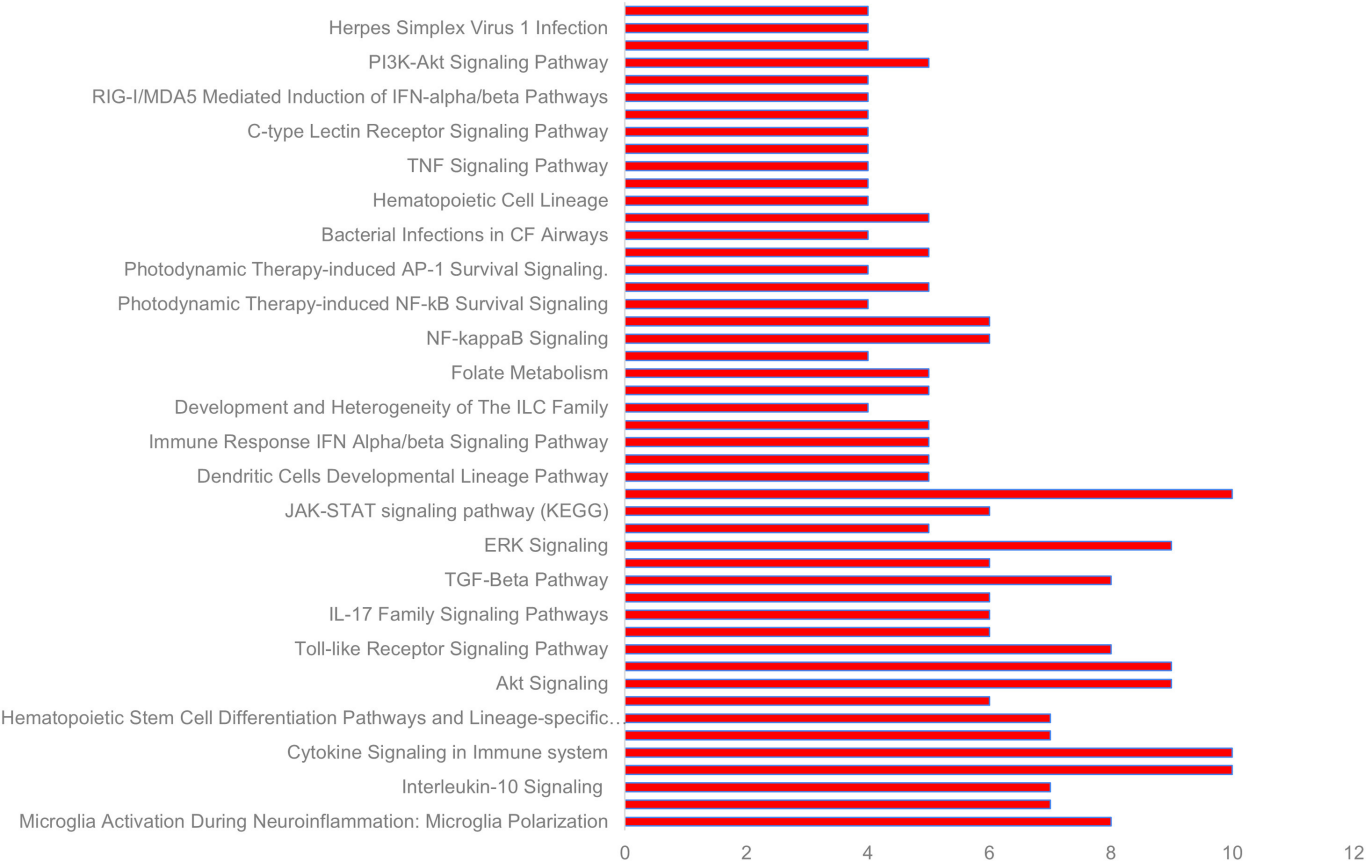
Some of the pathways associated with cytokines significant to COVID-19 cytokine storm (see Supplementary Table 3 for complete list).

GeneAnalytics mapped 10 out of 10 significant cytokines and 19 out of 21 lncRNAs with official gene names. Forty eight pathways were found to be associated with the 10 cytokines, including numerous molecular, cellular and disease pathways (Figure 4, Supplementary Table 3). All of the significant cytokines were involved in three pathways: PEDF induced signaling, cytokine signaling in immune system, and innate immune system. All significant cytokines except for IFNγ are also involved in the pathways: Akt signaling, PAK pathway, and ERK signaling.

Fifteen biological processes, 2 cellular components and 1 molecular function gene ontology terms were identified to be associated with the 10 significant cytokines (Table 4). All of the significant cytokines correlated with four gene ontology terms: cytokine activity, immune response, extracellular space, and extracellular region. One biological process, cytokine-mediated signaling pathway, is correlated with all significant cytokines except for IFNγ. Twenty cancer related diseases were found to be associated with the identified lncRNAs (Table 5), The cancers identified are not localized, and span across multiple organ systems. The disease with the most hits, hepatocellular carcinoma, is associated with ten out of nineteen mapped lncRNAs.

**Table 4:** Gene ontology terms associated with cytokines significant to COVID-19 cytokine storm.

**GO term**	**GEO ID**	**Ontology type**	**Number of cytokines**	**Cytokines**	**GeneAnalytics score**
Cytokine activity	GO:0005125	Molecular function	10	IL-10, IL-2, CSF3, IL-7, CCL2, CXCL10, IL-6, IFNγ, CCL3, TNFα	66.06
Immune response	GO:0006955	Biological process	10	IL-10, IL-2, CSF3, IL-7, CCL2, CXCL10, IL-6, IFNγ, CCL3, TNFα	53
Extracellular space	GO:0005615	Cellular component	10	IL-10, IL-2, CSF3, IL-7, CCL2, CXCL10, IL-6, IFNγ, CCL3, TNFα	34.8
Extracellular region	GO:0005576	Cellular component	10	IL-10, IL-2, CSF3, IL-7, CCL2, CXCL10, IL-6, IFNγ, CCL3, TNFα	30.94
Cytokine-mediated signaling pathway	GO:0019221	Biological process	9	IL-10, IL-2, CSF3, IL-7, CCL2, CXCL10, IL6, CCL3, TNFα	52
Signal transduction	GO:0007165	Biological process	7	IL-10, CSF3, IL-7, CCL2, CXCL10, IL-6, CCL3	16.95
Cellular response to lipopolysaccharide	GO:0071222	Biological process	6	IL-10, CSF3, CCL2, CXCL10, IL-6, TNFα	33.47
Inflammatory response	GO:0006954	Biological process	6	IL-10, CCL2, CXCL10, IL-6, CCL3, TNFα	26.78
Positive regulation of cell proliferation	GO:0008284	Biological process	6	IL-2, CSF3, IL-7, CXCL10, IL-6, IFNγ	23.94
Positive regulation of transcription By RNA polymerase II	GO:0045944	Biological process	6	IL-10, IL-2, CSF3, CXCL10, IL-6, TNFα	17.63
Humoral immune response	GO:0006959	Biological process	5	IL-7, CCL2, IL-6, IFNγ, TNFα	34.58
Growth factor activity	GO:0008083	Molecular function	5	IL-10, IL-2, CSF3, IL-7, IL-6	26.99
Positive regulation of gene expression	GO:0010628	Biological process	5	IL-7, IL-6, IFNγ, CCL3, TNFα	19.62
Positive regulation of tyrosine Phosphorylation of STAT protein	GO:0042531	Biological process	4	IL-2, IL-6, IFNγ, TNFα	24.91
Positive regulation of DNA-binding transcription factor activity	GO:0051091	Biological process	4	IL-10, CSF3, IL-6, TNFα	22.41
Cell-cell signaling	GO:0007267	Biological process	4	IL-2, IL-7, CXCL10, CCL3	18.44
MAPK cascade	GO:0000165	Biological process	4	IL-2, CCL2, CCL3, TNFα	17.8
Negative regulation of apoptotic process	GO:0043066	Biological process	4	IL-10, IL-2, IL-7, IL-6	13.44

**Table 5:** Diseases associated with identified lncRNAs in COVID-19 cytokine storm.

**Disease**	**Number of hits**	**lncRNAs**	**GeneAnalytics score**
Hepatocellular carcinoma	10	BANCR, CASC15, CRDNE, DRAIC, GAS5, MALAT1, NORAD, PRC1-AS1, SNHG1, TUG1	12.21
Gastric cancer	7	BANCR, CASC15, CRDNE, DRAIC, GAS5, MALAT1, SNHG1, TUG1	8.95
Colorectal cancer	7	BANCR, CRDNE, GAS5, MALAT1, NORAD, SNHG1, TUG1	8.29
Breast cancer	7	CRDNE, DRAIC, GAS5, MALAT1, NORAD, STXBP5-AS1, TUG1	8.22
Bladder cancer	6	BANCR, DRAIC, GAS5, MALAT1, NORAD, TUG1	8.39
Lung cancer	6	BANCR, GAS5, MALAT1, SBF2-AS1, SNHG1, TUG1	7.13
Osteogenic sarcoma	5	BANCR, GAS5, MALAT1, SNHG1, TUG1	7.64
Melanoma	5	BANCR, GAS5, MALAT1, SBF2-AS1, SNHG1, TUG1	6.9
Esophageal cancer	5	GAS5, MALAT1, NORAD, SBF2-AS1, SNHG1	6.83
Pancreatic cancer	5	CRDNE, GAS5, MALAT1, NORAD, TUG1	6.65
Prostate cancer	5	DRAIC, GAS5, MALAT1, SNHG1, TUG1	6.03
Thyroid cancer, Non-medullary 1	4	BANCR, CRDNE, GAS5, MALAT1	7.17
Malignant glioma	4	CRDNE, GAS5, MALAT1, TUG1	6.39
Bladder urothelial carcinoma	4	CRDNE, GAS5, MALAT1, TUG1	6.1
Astrocytoma	4	CASC15, CSK6-AS1, GAS5, SNHG1	5.61
Neuroblastoma	4	CASC15, GAS5, MALAT1, SNHG1	5.46
Cervical cancer	4	CRDNE, GAS5, MALAT1, TUG1	5.24
Renal cell carcinoma, Non-papillary	4	CRDNE, GAS5, MALAT1, TUG1	5.17
Lung cancer susceptibility 3	4	DRAIC, GAS5, MALAT1, TUG1	5.11
Ovarian cancer	4	CRDNE, GAS5, MALAT1, TUG1	5

### Evolutionary Trace of lncRNA NORAD and Stable Folding

**Figure 5:**
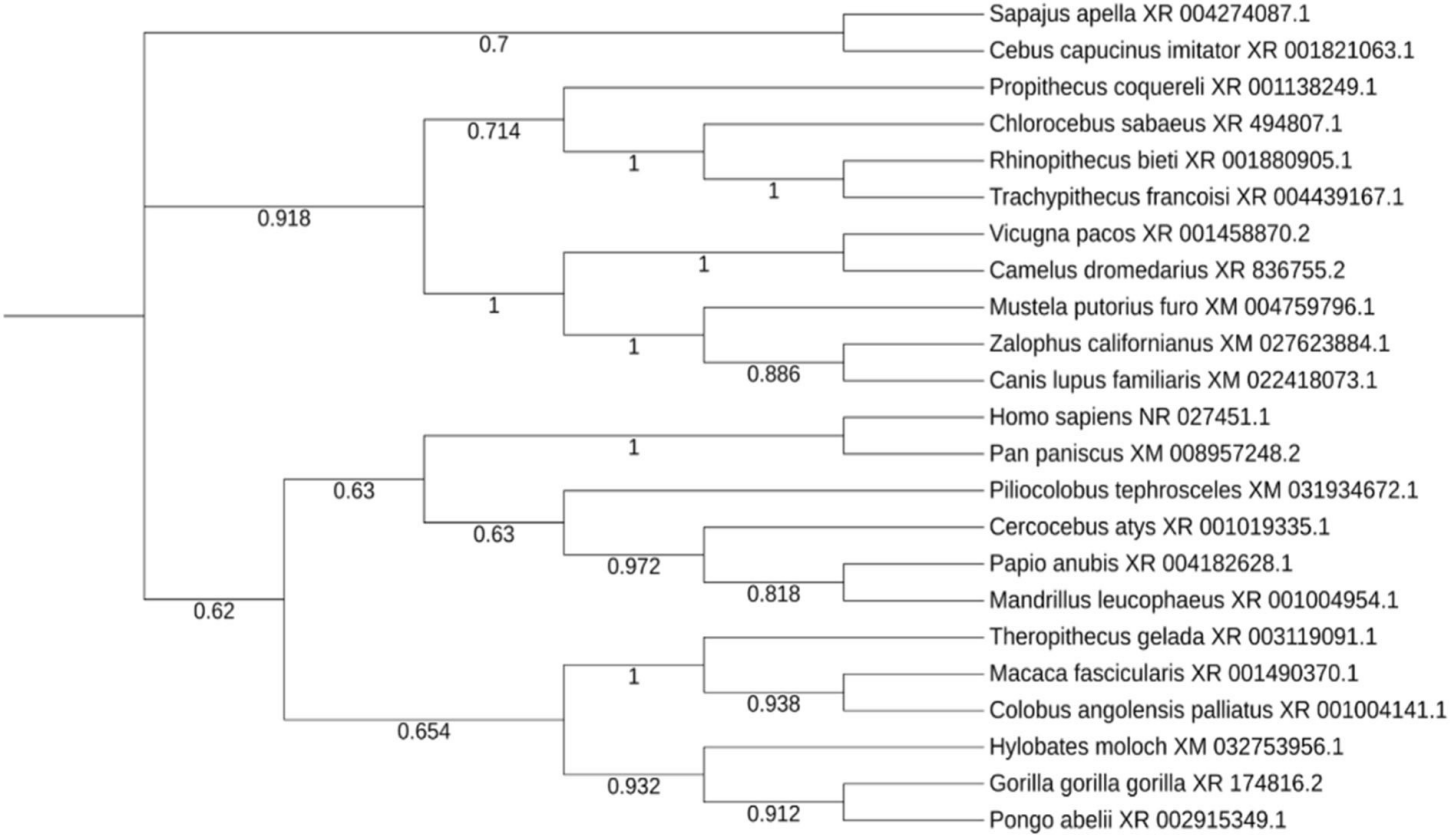
Evolutionary trace of lncRNA NORAD in 23 related species, with bootstrap values at each node.

**Figure 6:**
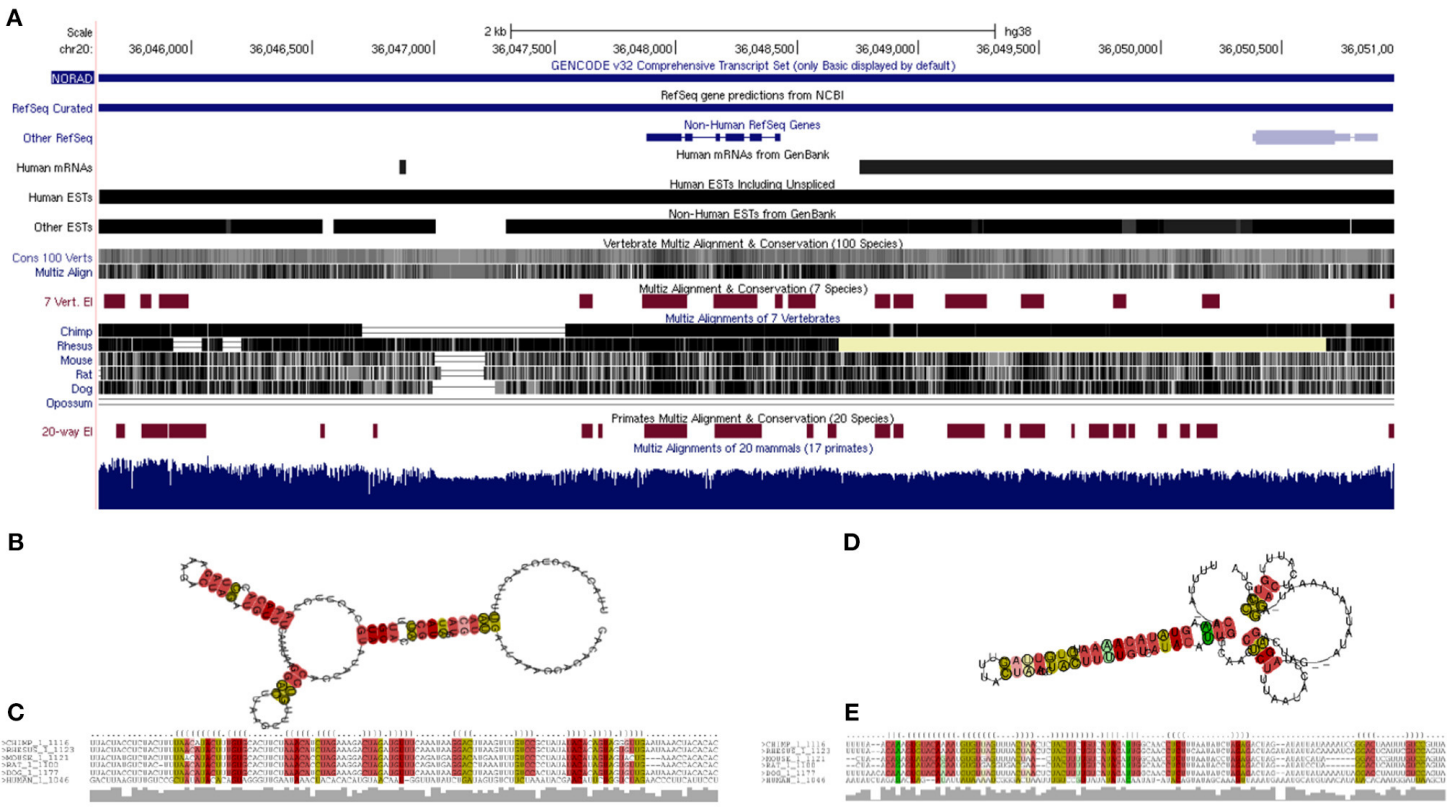
Annotated reference sequence of human lncRNA NORAD, its genomic and expressed sequence tags (ESTs) conservation across the mammalian genome **(A)**; thermodynamically stable and evolutionarily conserved RNA-fold from multiple sequence alignment of six mammalian NORAD genomic sequences with probabilities of 0.92 **(B)** and 1.0 **(D)**, respectively; multiple sequence alignment of six mammalian NORAD genomic sequences used for predicting secondary structures; colors show mutational pattern with respect to the structure **(C,E)**. Pale color indicates a base pair cannot be found in some alignment.

Twenty three unique genera were represented in the top 100 results from a NCBI BLASTn search using the lncRNA NORAD. For genera with multiple species represented in the results, the species with the max score was selected for phylogenetic analysis. A phylogenetic tree, labeled by species and accession number was created and visualized (Figure 5). There are three major ingroups, though two of the three ingroups have moderate bootstrap values (0.7 and 0.654). The ingroup with the *Homo sapiens* NORAD sequence consists entirely of primates. The *Homo sapiens* NORAD sequence was most phylogenetically related to another lncRNA found in *Pan paniscus*, with a bootstrap value of 1. The 23 sequences represented in the tree are not exclusively from primates; 6 of the 9 sequences in the ingroup shown in black are from non-primate species. Figure 6A depicts annotated NORAD conserved regions across several mammalian genomic sequences and ESTs from the UCSC genome browser, while Figures 6B–E show two representation of significantly conserved RNA folding and multiple sequence alignment among six mammals (human, chimpanzee, monkey, rat, mouse and dog) with *p*-values of 0.92 and 1.0, respectively. A *p*-value above 0.5 shows that lncRNA NORAD has a significantly high evolutionary conservation among these mammalian genomes and that the higher folding strength of NORAD is above threshold, an additional evidence of evolutionary conservation.

## Discussion

SARS-CoV-2 is a novel pathogen that has evolved into a pandemic, with significant mortality and morbidity rates. With millions infected, it is imperative to develop a better understanding of its pathology in order to develop treatments. An indicator for increased COVID-19 severity is cytokine storm, of which its genetic regulatory elements are poorly understood. We identified lncRNAs that can target significant cytokines during COVID-19 cytokine storm with computational tools. Ten cytokines significant in COVID-19 cytokine storm were selected out of a review of 17 papers for further analysis (as of May 2020). The cytokine IL-6 was identified to be significant in 13 out of 17 papers reviewed. IL-6 is currently being investigated as a potential drug target, with clinical trials underway for the IL-6 antagonist tocilizumab (Guaraldi et al., 2020; Kewan et al., 2020; Liu et al., 2020; Xu et al., 2020). A total of 22 lncRNAs were identified to bind with the 10 significant cytokines, all with binding free energies <-0.02. The lncRNA NORAD targeted 5 of the 10 significant cytokines, while the lncRNAs RAD51-AS1 and lnrCXCR4 each target three of the significant cytokines. Since lncRNAs are known to contribute to transcriptional and epigenetic regulation as well as post transcriptional modifications, we hypothesize that lncRNAs that are able to target and bind to significant cytokine nucleotide sequences have the potential to downregulate cytokine expression, which can ameliorate the pro-inflammatory immune response to COVID-19 infection, mitigating cytokine storm in the process. Potential translational approaches to administering this technology in the clinical setting include antisense oligonucleotides knockdown, RNAi knockdown, and viral gene therapy (Fatemi et al., 2014; Lennox and Behlke, 2016; Roberts et al., 2020).

NORAD targeted five of the ten cytokines involved in cytokine storm, more than any other identified lncRNA. NORAD, short for non-coding RNA activated by DNA damage, is responsible for chromosome stability and mitotic division (Lee et al., 2016; Tichon et al., 2016). Upregulation of NORAD is associated with 6 different types of cancers, and overexpression of NORAD leads to poor overall survival in cancer patients (Yang et al., 2019). In the context of SARS-CoV-2 viral infection, upregulation of NORAD may be a response to aberrant viral nucleotide replication within macrophages.

RAD51-AS1 is shown to inhibit DNA repair, and has a conserved E2F1 binding site in its promoter region (Zhang et al., 2017). RAD51-AS1 has been shown to inhibit DNA damage repair ability in hepatocellular carcinoma cells. E2F1 is a transcription factor that regulates the cell cycle and apoptosis (Qin et al., 1994; Shan and Lee, 1994; Wu and Levine, 1994). RAS51-AS1 may be expressed in the context of viral replication within macrophages. RAD51-AS1 has also been proposed as a prognostic marker for epithelial ovarian cancer (Zhang et al., 2017). In the context of SARS-CoV-2 infection, RAD51-AS1 may be expressed in response to cellular damage from viral replication within macrophages, leading to expression of pro-inflammatory cytokines. The lncRNA lnrCRCX4 has an NCBI GEO entry, but does not have any associated published literature.

The lncRNAs SBF2-AS1, TUG1, GAS5, SNHG1, and NRAV all target two of the significant cytokines. SBF2-AS1 has been proposed as a disease marker for *A. fumigatus* (Riege et al., 2017). TUG1 knockdown has been found to decrease inflammatory response in atherosclerotic lesions (Zhang H. et al., 2018). In contrast, TUG1 overexpression results in decreased levels of pro-apoptotic factors and inflammation in lipopolysaccharide exposed H9c2 cells cytokines (Zhang L. et al., 2018). GAS5 has also been shown to inhibit NF-κB and Notch signaling pathways and reduce lipopolysaccharide inflammatory injury in ATDC5 chondrocytes (Li et al., 2018). SNHG1 upregulation has been found to significantly decrease the production of pro-inflammatory cytokines–NO, PGE2, IL-6, and TNFα in human chondrocytes (Lei et al., 2019). These lncRNAs have been demonstrated to have the potential to be disease markers and negative regulators of pro-inflammatory cytokines.

Notably NRAV, abbreviated for negative regulation of antiviral response, is shown to downregulate interferon stimulating genes (ISG) (Ouyang et al., 2014). Silencing NRAV has been shown to suppress influenza A virus replication and virulence. Interestingly, the receptor for COVID-19, ACE2, is an ISG (Ziegler et al., 2020). Because SARS-CoV-2 must balance aggravating the host immune response and promoting infectivity, the expression levels of NRAV would be an interesting lncRNA for future studies to characterize gene expression.

The metabolic pathways that involve 8 or more of the 10 significant cytokines; PEDF signaling, cytokine signaling in the immune system, innate immune system, Akt signaling, PAK pathway, and ERK signaling; are all related to an inflammatory response to a pathogen (Kurosawa et al., 2000; Yabe et al., 2005; Chan et al., 2007; Sun et al., 2018). Likewise, the gene ontology terms associated with the 10 significant cytokines are consistent with pro-inflammatory innate immune response. The diseases associated with the identified lncRNAs are cancers of multiple organ systems possibly because lncRNAs are tissue specific. The breadth of the organ systems affected may be linked to the systemic nature of cytokine storms. Cancers are a dysregulation of normal cellular processes resulting in uncontrolled growth; thus, it parallels with cytokine storm in that both are hyper-activations of normal cellular processes.

An evolutionary trace was performed on the lncRNA NORAD using 23 highly similar sequences from neighboring species. Despite lncRNAs as a whole being poorly conserved (Hezroni et al., 2015), NORAD shows a high degree of conservation among related species, especially in mammals. All of the sequences in the same branch of the tree (in blue) as the *Homo sapiens* NORAD sequence are primates. The species sharing the closest common ancestor, *Pan paniscus*, is paired with the *Homo sapiens* sequence with a bootstrap value of 1. This indicates that *Pan paniscus* is a promising model organism for further evaluating the role and pathology of NORAD in COVID-19 disease pathology, and potentially useful for testing new medications before clinical trials in humans. LncRNAs could fold in diverse complex manner to form secondary and varying functions. Our study reveal a strong evidence of conserved NORAD structural folding across mammalian genomes with a high thermodynamic stability, given its peculiar ability to interact or bind many cytokines. In addition, its high folding strength suggests a positive correlation with expression and functional significance. Taken together, these results provide evidence of significant evolutionary conservation and functional stability across mammalian genomes, further strengthening its candidacy for gene regulation during immune response to SARS-CoV-2 perturbation.

## Conclusion

In SARS-CoV-2 infection, no treatments are approved to treat cytokine storm, a precursor to ARDS. We sought to computationally identify cytokines that were significantly upregulated in COVID-19 cytokine storm, and lncRNAs that can target these cytokines. From literature review, we found 10 cytokines to be significantly upregulated in COVID-19 cytokine storm and were selected for further analysis. We identified 22 lncRNAs that can target these cytokines, 8 of which can target multiple cytokines. Of particular and possibly clinical importance, we report that lncRNA NORAD can target 5 of the 10 significant cytokines. Though lncRNAs are known to be less conserved across species, conversely and of interest, we found NORAD to be highly conserved across multiple mammalian species, in addition to previous reports of its overexpression associated with multiple cancer phenotypes. Additionally, 5 lncRNAs that target multiple cytokines have been experimentally identified to have roles in inflammatory responses. Therefore, these lncRNAs show potential as targets for intervention during SARS-CoV-2 pathogenesis, and are prime candidates for further *in vivo* and *in vitro* analysis.

## Data Availability Statement

The original contributions presented in the study are included in the article/Supplementary Material, further inquiries can be directed to the corresponding author/s.

## Author Contributions

OM and BT conceptualized and designed the experiments. OM carried out the experiments, analyzed the data, and drafted the manuscript. KB, ES, and MW contributed to the data analysis, manuscript draft and scientific content. OM and BT revised the manuscript, contributed to the discussion and scientific content. All authors read and approved the final version of the manuscript.

## Conflict of Interest

The authors declare that the research was conducted in the absence of any commercial or financial relationships that could be construed as a potential conflict of interest.
