# Supplementary material for: Evolutionarily Conserved Long Non-coding RNA Regulates Gene Expression in Cytokine Storm During COVID-19
Source: Front Bioeng Biotechnol. 2021 Jan 15;8:582953. doi: 10.3389/fbioe.2020.582953 (PMC7844208; doi:10.3389/fbioe.2020.582953)
Supplement: Supplementary file 1 [file Table_1.DOCX]

**Supplementary Table 1. Research articles referenced to find cytokines correlated with COVID-19 disease severity and/or cytokine storm**

| **Research Article:** | **Link:** | **Number of cytokines** |
| --- | --- | --- |
| Chen et al. 2020 | [Clinical and immunologic features in severe and moderate Coronavirus Disease 2019](https://www.jci.org/articles/view/137244) | 1 |
| Chen et al. 2020 | [Epidemiological and clinical characteristics of 99 cases of 2019 novel coronavirus pneumonia in Wuhan, China: a descriptive study](https://www.sciencedirect.com/science/article/pii/S0140673620302117?via%3Dihub) | 2 |
| Chen et al. 2020 | [Detectable Serum SARS-CoV-2 Viral Load (RNAaemia) is Closely Associated With Drastically Elevated Interleukin 6 (IL-6) Level in Critically Ill COVID-19 Patients](https://www.medrxiv.org/content/10.1101/2020.02.29.20029520v1) | 14 |
| Chu et al. 2020 | [Comparative replication and immune activation profiles of SARS-CoV-2 and SARS-CoV in human lungs: an ex vivo study with implications for the pathogenesis of COVID-19](https://academic.oup.com/cid/advance-article/doi/10.1093/cid/ciaa410/5818134) | 3 |
| Diao et al. 2020 | [Reduction and Functional Exhaustion of T Cells in Patients With Coronavirus Disease 2019 (COVID-19)](https://www.medrxiv.org/content/10.1101/2020.02.18.20024364v1) | 1 |
| Huang et al. 2020 | [Clinical features of patients infected with 2019 novel coronavirus in Wuhan, China](https://www.sciencedirect.com/science/article/pii/S0140673620301835) | 5 |
| Liu et al. 2020 | [2019-novel Coronavirus (2019-nCoV) Infections Trigger an Exaggerated Cytokine Response Aggravating Lung Injury](http://www.chinaxiv.org/abs/202002.00018) | 3 |
| Mehta et al. 2020 | [HLH Across Speciality Collaboration, UK, COVID-19: consider cytokine storm syndromes and immunosuppression](https://www.sciencedirect.com/science/article/pii/S0140673620306280) | 1 |
| Qin et al. 2020 | [Dysregulation of immune response in patients with COVID-19 in Wuhan, China](https://academic.oup.com/cid/advance-article/doi/10.1093/cid/ciaa248/5803306) | 3 |
| Ruan et al. 2020 | [Clinical predictors of mortality due to COVID-19 based on an analysis of data of 150 patients from Wuhan, China](https://link.springer.com/article/10.1007%2Fs00134-020-05991-x) | 1 |
| Sun et al. 2020 | [Clinical features of severe pediatric patients with coronavirus disease 2019 in Wuhan: a single center’s observational study](https://link.springer.com/article/10.1007%2Fs12519-020-00354-4) | 2 |
| Wan et al. 2020 | [Characteristics of lymphocyte subsets and cytokines in peripheral blood of 123 hospitalized patients with 2019 novel coronavirus pneumonia (NCP)](https://www.medrxiv.org/content/10.1101/2020.02.10.20021832v1) | 14 |
| Wang et al. 2020 | [The definition and risks of cytokine release syndrome-like in 11 COVID-19-Infected pneumonia critically ill patients: disease characteristics and retrospective analysis](https://www.medrxiv.org/content/10.1101/2020.02.26.20026989v1) | 8 |
| Wu et al 2020 | [Risk Factors Associated With Acute Respiratory Distress Syndrome and Death in Patients With Coronavirus Disease 2019 Pneumonia in Wuhan, China](https://jamanetwork.com/journals/jamainternalmedicine/fullarticle/2763184) | 7 |
| Xiong et al. 2020 | [Transcriptomic characteristics of bronchoalveolar lavage fluid and peripheral blood mononuclear cells in COVID-19 patients](https://www.tandfonline.com/doi/full/10.1080/22221751.2020.1747363) | 4 |
| Yang et al. 2020 | [Exuberant elevation of IP-10, MCP-3 and IL-1ra during SARS-CoV-2 infection is associated with disease severity and fatal outcome](https://www.medrxiv.org/content/10.1101/2020.03.02.20029975v1) | 1 |
| Zhou et al. 2020 | [Aberrant pathogenic GM-CSF+ T cells and inflammatory CD14+CD16+ monocytes in severe pulmonary syndrome patients of a new coronavirus](https://www.biorxiv.org/content/10.1101/2020.02.12.945576v1) | 1 |
