# Supplementary material for: Evolutionarily Conserved Long Non-coding RNA Regulates Gene Expression in Cytokine Storm During COVID-19
Source: Front Bioeng Biotechnol. 2021 Jan 15;8:582953. doi: 10.3389/fbioe.2020.582953 (PMC7844208; doi:10.3389/fbioe.2020.582953)
Supplement: Supplementary file 2 [file Table_2.DOCX]

**Supplementary Table 2. Binding free energies (ndG) for the lncRNAs and their associated cytokine gene targets**

| **Target** | **Length_Target** | **Query** | **ndG** |
| --- | --- | --- | --- |
| IL-2 | 814 | BANCR | -0.0341 |
|  |  | lnrCXCR4 | -0.0387 |
|  |  | DRAIC | -0.0448 |
| IL-6 | 1127 | lnc-IL7R | -0.0324 |
|  |  | LNCSRLR | -0.0343 |
|  |  | SBF2-AS1 | -0.0327 |
|  |  | RAD51-AS1 | -0.0469 |
|  |  | NORAD | -0.0493 |
|  |  | SENCR | -0.0383 |
|  |  | LNC-LCBS_1 | -0.0361 |
|  |  | LNC-LBCS_2 | -0.0326 |
|  |  | LNC-LBCS_3 | -0.0374 |
|  |  | LNC-LBCS_4 | -0.0335 |
|  |  | LNC-LBCS_5 | -0.049 |
| IL-7 | 2125 | TUG1 | -0.0949 |
|  |  | SBF2-AS1 | -0.0398 |
| IL-10 | 691 | GAS5 | -0.0935 |
|  |  | lnrCXCR4 | -0.0668 |
|  |  | NORAD | -0.0637 |
|  |  | SNHG1 | -0.0436 |
| CSF3 | 1671 | NORAD | -0.0505 |
|  |  | lincSTXBP5_1 | -0.0848 |
|  |  | lincSTXBP5_2 | -0.0407 |
|  |  | lincSTXBP5_3 | -0.0461 |
|  |  | lincSTXBP5_4 | -0.0384 |
|  |  | lincSTXBP5_5 | -0.0341 |
|  |  | lincSTXBP5_6 | -0.0382 |
|  |  | lincSTXBP5_7 | -0.0387 |
|  |  | lincSTXBP5_8 | -0.0444 |
|  |  | lincSTXBP5_9 | -0.0435 |
|  |  | lincSTXBP5_10 | -0.0468 |
|  |  | lincSTXBP5_11 | -0.0437 |
|  |  | lincSTXBP5_12 | -0.0389 |
|  |  | lincSTXBP5_13 | -0.043 |
|  |  | lincSTXBP5_14 | -0.0458 |
|  |  | lincSTXBP5_15 | -0.0487 |
|  |  | lincSTXBP5_16 | -0.042 |
|  |  | lincSTXBP5_17 | -0.0425 |
|  |  | lincSTXBP5_18 | -0.0442 |
|  |  | lincSTXBP5_19 | -0.0386 |
|  |  | lincSTXBP5_20 | -0.0563 |
|  |  | lincSTXBP5_21 | -0.0493 |
| TNFα | 1676 | THRIL | -0.0416 |
|  |  | RAD51-AS1 | -0.0432 |
|  |  | CASC15 | -0.0323 |
|  |  | NORAD | -0.0502 |
|  |  | GAS5 | -0.0824 |
|  |  | NRCP | -0.0319 |
|  |  | CASC15 | -0.0323 |
| IFNγ | 1244 | TMEVPG1 | -0.0222 |
|  |  | PRC1-AS1 | -0.0292 |
| CXCL10 | 1207 | NORAD | -0.0319 |
| CCL2 | 743 | MALAT1 | -0.0527 |
|  |  | TUG1 | -0.0543 |
|  |  | RAD51-AS1 | -0.0433 |
|  |  | SNHUG1 | -0.0342 |
|  |  | NRAV | -0.0449 |
| CCL3 | 784 | lnrCXCR4 | -0.0926 |
|  |  | NRAV | -0.0633 |
|  |  | CDK6-AS1_1 | -0.0676 |
|  |  | CDK6-AS1_2 | -0.0521 |
|  |  | CDK6-AS1_3 | -0.0546 |
|  |  | CDK6-AS1_4 | -0.0941 |
|  |  | CDK6-AS1_5 | -0.048 |
