# Supplementary material for: Evolutionarily Conserved Long Non-coding RNA Regulates Gene Expression in Cytokine Storm During COVID-19
Source: Front Bioeng Biotechnol. 2021 Jan 15;8:582953. doi: 10.3389/fbioe.2020.582953 (PMC7844208; doi:10.3389/fbioe.2020.582953)
Supplement: Supplementary file 3 [file Table_3.DOCX]

**Supplementary Table 3. Pathways associated with cytokines significant to COVID-19 cytokine storm**

| **Pathway** | **Number of Cytokines** | **Cytokines** | **GeneAnalytics Score** |
| --- | --- | --- | --- |
| PEDF Induced Signaling | 10 | IL-10, IL-2, CSF3, IL-7, CCL2, CXCL10, IL-6, IFNγ, CCL3, TNFα | 48.05 |
| Cytokine Signaling in Immune system | 10 | IL-10, IL-2, CSF3, IL-7, CCL2, CXCL10, IL-6, IFNγ, CCL3, TNFα | 47.71 |
| Innate Immune System | 10 | IL-10, IL-2, CSF3, IL-7, CCL2, CXCL10, IL-6, IFNγ, CCL3, TNFα | 32.88 |
| Akt Signaling | 9 | IL-10, IL-2, CSF3, IL-7, CCL2, CXCL10, IL-6, CCL3, TNFα | 41.07 |
| PAK Pathway | 9 | IL-10, IL-2, CSF3, IL-7, CCL2, CXCL10, IL-6, CCL3, TNFα | 41.05 |
| ERK Signaling | 9 | IL-10, IL-2, CSF3, IL-7, CCL2, CXCL10, IL-6, CCL3, TNFα | 33.99 |
| Microglia Activation During Neuroinflammation: Microglia Polarization | 8 | IL-10, IL-2, CCL2, CXCL10, IL-6, IFNγ, CCL3, TNFα | 60.05 |
| Toll-like Receptor Signaling Pathway | 8 | IL-10, IL-2, CCL2, CXCL10, IL-6, IFNγ, CCL3, TNFα | 40.72 |
| TGF-Beta Pathway | 8 | IL-10, IL-2, IL-7, CCL2, IL-6, IFNγ, CCL3, TNFα | 34.51 |
| Cytokines and Inflammatory Response | 7 | IL-10, IL-2, CSF3, IL-7, IL-6, IFNγ, TNFα | 59.48 |
| Interleukin-10 Signaling | 7 | IL-10, CSF3, CCL2, CXCL10, IL-6, CCL3, TNFα | 54.19 |
| Chemokine Superfamily Pathway: Human/Mouse Ligand-Receptor Interactions | 7 | IL-10, IL-2, CCL2, CXCL10, IL-6, CCL3, TNFα | 46.31 |
| Hematopoietic Stem Cell Differentiation Pathways and Lineage-specific Markers | 7 | IL-10, IL-2, CSF3, IL-7, IL-6, IFNγ, TNFα | 45.49 |
| Malaria | 6 | IL-10, CSF3, CCL2, IL-6, IFNγ, TNFα | 44.48 |
| Innate Lymphoid Cell Differentiation Pathways | 6 | IL-10, IL-2, IL-7, IFNγ, CCL3, TNFα | 40.42 |
| IL-17 Family Signaling Pathways | 6 | CSF3, CCL2, CXCL10, IL-6, IFNγ, TNFα | 37.21 |
| Spinal Cord Injury | 6 | IL2, CCL2, CXCL10, IL6, IFNγ, TNFα | 36.92 |
| Immune response IL-23 signaling pathway | 6 | IL-10, IL-2, CCL2, IL-6, IFNγ, TNFα | 34.38 |
| JAK-STAT signaling pathway (KEGG) | 6 | IL-10, IL-2, CSF3, IL-7, IL-6, IFNγ | 33.86 |
| NF-κB Signaling | 6 | IL-10, IL-2, CCL2, CXCL10, IFNγ, TNFα | 28.29 |
| Measles | 6 | IL-2, CCL2, CXCL10, IL-6, IFNγ, TNFα | 28.26 |
| Lung fibrosis | 5 | CSF3, CCL2, IL-6, CCL3, TNFα | 33.86 |
| Dendritic Cells Developmental Lineage Pathway | 5 | IL-10, IL-2, IL-6, IFNγ, TNFα | 32.7 |
| Th1 Differentiation Pathway | 5 | IL-10, IL-2, IL-6, IFNγ, TNFα | 32.32 |
| Immune Response IFN Alpha/beta Signaling Pathway | 5 | IL-10, IL-2, IL-6, IFNγ, TNFα | 32.21 |
| Rheumatoid Arthritis | 5 | CCL2, IL-6, IFNγ, CCL3, TNFα | 31.06 |
| Yersinia Infection | 5 | IL-10, IL-2, CCL2, IL-6, TNFα | 29.23 |
| Folate Metabolism | 5 | IL-2, CCL2, IL-6, IFNγ, TNFα | 29.23 |
| Th17 Cell Differentiation | 5 | IL-10, IL-2, IL-6, IFNγ, TNFα | 27.08 |
| T Cell Receptor Signaling Pathway | 5 | IL-10, IL-2, IL-6, IFNγ, TNFα | 26.2 |
| Allograft Rejection | 5 | IL-10, IL-2, IL6, IFNγ, TNFα | 23.97 |
| PI3K-Akt Signaling Pathway | 5 | IL-2, CSF3, IL-7, IL-6, TNFα | 18.71 |
| Development and heterogeneity of ILC Family | 4 | IL-7, IL-6, IFNγ, TNFα | 29.66 |
| African Trypanosomiasis | 4 | IL-10, IL-6, IFNγ, TNFα | 28.82 |
| Photodynamic Therapy-induced NF-kB Survival Signaling | 4 | IL-2, IL-6, IFNγ, TNFα | 27.33 |
| Photodynamic Therapy-induced AP-1 Survival Signaling. | 4 | IL-2, IL-6, IFNγ, TNFα | 26.98 |
| Bacterial Infections in CF Airways | 4 | CXCL10, IL-6, IFNγ, TNFα | 25.07 |
| Hematopoietic Cell Lineage | 4 | CSF3, IL-7, IL-6, TNFα | 23.16 |
| Amoebiasis | 4 | IL-10, IL-6, IFNγ, TNFα | 22.99 |
| TNF Signaling Pathway | 4 | CCL2, CXCL10, IL-6, TNF | 22.46 |
| Interleukin-4 and 13 Signaling | 4 | IL-10, CCL2, IL-6, TNFα | 22.36 |
| C-type Lectin Receptor Signaling Pathway | 4 | IL-10, IL-2, IL6, TNFα | 21.74 |
| Common Cytokine Receptor Gamma-Chain Family Signaling Pathways | 4 | IL-2, IL-7, IL-6, TNFα | 21.1 |
| RIG-I/MDA5 Mediated Induction of IFN-alpha/beta Pathways | 4 | CXCL10, IL-6, IFNγ, TNFα | 20.45 |
| Tuberculosis | 4 | IL-10, IL-6, IFNγ, TNFα | 19.74 |
| Human Cytomegalovirus Infection | 4 | CCL2, IL-6, CCL3, TNFα | 15.8 |
| Herpes Simplex Virus 1 Infection | 4 | CCL2, IL-6, IFNγ, TNFα | 14.06 |
| Pathways in Cancer | 4 | IL-2, IL-7, IL-6, IFNγ | 13.62 |
